# Basiliximab impairs regulatory T cell (TREG) function and could affect the short-term graft acceptance in children with heart transplantation

**DOI:** 10.1038/s41598-020-80567-9

**Published:** 2021-01-12

**Authors:** Jacobo López-Abente, Marta Martínez-Bonet, Esther Bernaldo-de-Quirós, Manuela Camino, Nuria Gil, Esther Panadero, Juan Miguel Gil-Jaurena, Maribel Clemente, Simon Urschel, Lori West, Marjorie Pion, Rafael Correa-Rocha

**Affiliations:** 1 Laboratory of Immune-Regulation, Instituto de Investigación Sanitaria Gregorio Marañón (IiSGM), Pabellón de Medicina Experimental, Planta Baja. C/ Maiquez, 6., 28006 Madrid, Spain; 2 Pediatric-Cardiology Department, Hospital General Universitario Gregorio Marañón, Madrid, Spain; 3 Pediatric Cardiac Surgery Unit, Hospital General Universitario Gregorio Marañón, Madrid, Spain; 4 Cell Culture Unit, Instituto de Investigación Sanitaria Gregorio Marañón (IiSGM), Madrid, Spain; 5 Pediatric Cardiac Transplantation, University of Alberta/Stollery Children’s Hospital, Edmonton, AB Canada; 6 Alberta Transplant Institute, University of Alberta, Edmonton, AB Canada; 7 Canadian National Transplant Research Program Investigator, CNTRP, Edmonton, AB Canada

**Keywords:** Immunology, Peripheral tolerance, Paediatric research, Immunosuppression

## Abstract

CD25, the alpha chain of the IL-2 receptor, is expressed on activated effector T cells that mediate immune graft damage. Induction immunosuppression is commonly used in solid organ transplantation and can include antibodies blocking CD25. However, regulatory T cells (Tregs) also rely on CD25 for their proliferation, survival, and regulatory function. Therefore, CD25-blockade may compromise Treg protective role against rejection. We analysed in vitro the effect of basiliximab (BXM) on the viability, phenotype, proliferation and cytokine production of Treg cells. We also evaluated in vivo the effect of BXM on Treg in thymectomized heart transplant children receiving BXM in comparison to patients not receiving induction therapy. Our results show that BXM reduces Treg counts and function in vitro by affecting their proliferation, Foxp3 expression, and IL-10 secretion capacity. In pediatric heart-transplant patients, we observed decreased Treg counts and a diminished Treg/Teff ratio in BXM-treated patients up to 6-month after treatment, recovering baseline values at the end of the 12-month follow up period. These results reveal that the use of BXM could produce detrimental effects on Tregs, and support the evidence suggesting that BXM induction could impair the protective role of Tregs in the period of highest incidence of acute graft rejection.

## Introduction

Many transplant programs employ induction immunosuppression**,** a relatively intense prophylactic therapy, at the time of transplantation based on the empiric observation that potent immunosuppression is required to prevent early acute rejection^1^. However, there remains controversy whether these agents are absolutely required and about their risk/benefit balance of their use^1^. Moreover, little is known about the specific effects of these immunosuppressants on certain immune cell subsets and the consequences for immune homeostasis.

Of induction medications, the IL-2 receptor antagonist currently utilized in the clinics is basiliximab (BXM), a therapeutic monoclonal antibody that reversibly binds the IL-2 receptor alpha chain (IL-2Ra or CD25) and can completely block its interaction with IL-2^2,3^. Its use aims to block activated effector T cells^4^; however, regulatory T cells (Tregs) also express high levels of CD25 constitutively^5^, and rely on CD25 not only for proliferation and survival but also for detection of excessive proliferation of effector cells^6,7^. In the context of transplantation, Tregs prevent activation and expansion of effector T cells implicated in cellular rejection and also induce B cell death, preventing humoral rejection, as observed in animal heart transplant models^8^. Therefore, Tregs play a crucial role in the regulation of immune processes essential for transplant acceptance^9,10^, and Tregs were shown to promote transplantation tolerance and indefinite allograft survival in renal transplant and animal models^11,12^. Indeed, the Treg to effector T-cell (Teff) balance was described to be crucial in development of either graft rejection or allograft tolerance^10^. Therefore, while BXM may prevent acute rejection by hindering IL-2-mediated Teff expansion, it could also compromise the mechanisms of graft acceptance by impairing Treg proliferation and function.

In the case of children requiring heart transplantation, BXM-mediated Treg impairment could be even more problematic. The thymus is typically removed for surgical field exposure in pediatric cardiac surgeries, making pediatric heart transplant recipients unable to regenerate the thymus-derived Treg population.

In this study, we investigated in vitro whether BXM had detrimental effects on Treg survival, proliferation and functionality compared to other T-cell populations. Additionally, we compared Treg values and their evolution in pediatric heart transplant patients with and without BXM induction therapy.

## Results

### In vitro BXM treatment has a direct effect on both Treg counts and Foxp3 expression

We analysed in vitro the effect of BXM on Treg from healthy donors. We studied the BXM effect on T cells after 72 h of culture and employing: (i) non-activating conditions, which could be indicative of the BXM effect on an immune system in a quiescent status; (ii) activation employing anti-CD3/CD28 coated beads, which mimic antigen-presenting cells and activate T cells and thus could be indicative of the BXM effect on an immune system activated by the presence of alloantigens.

**Figure 1:**
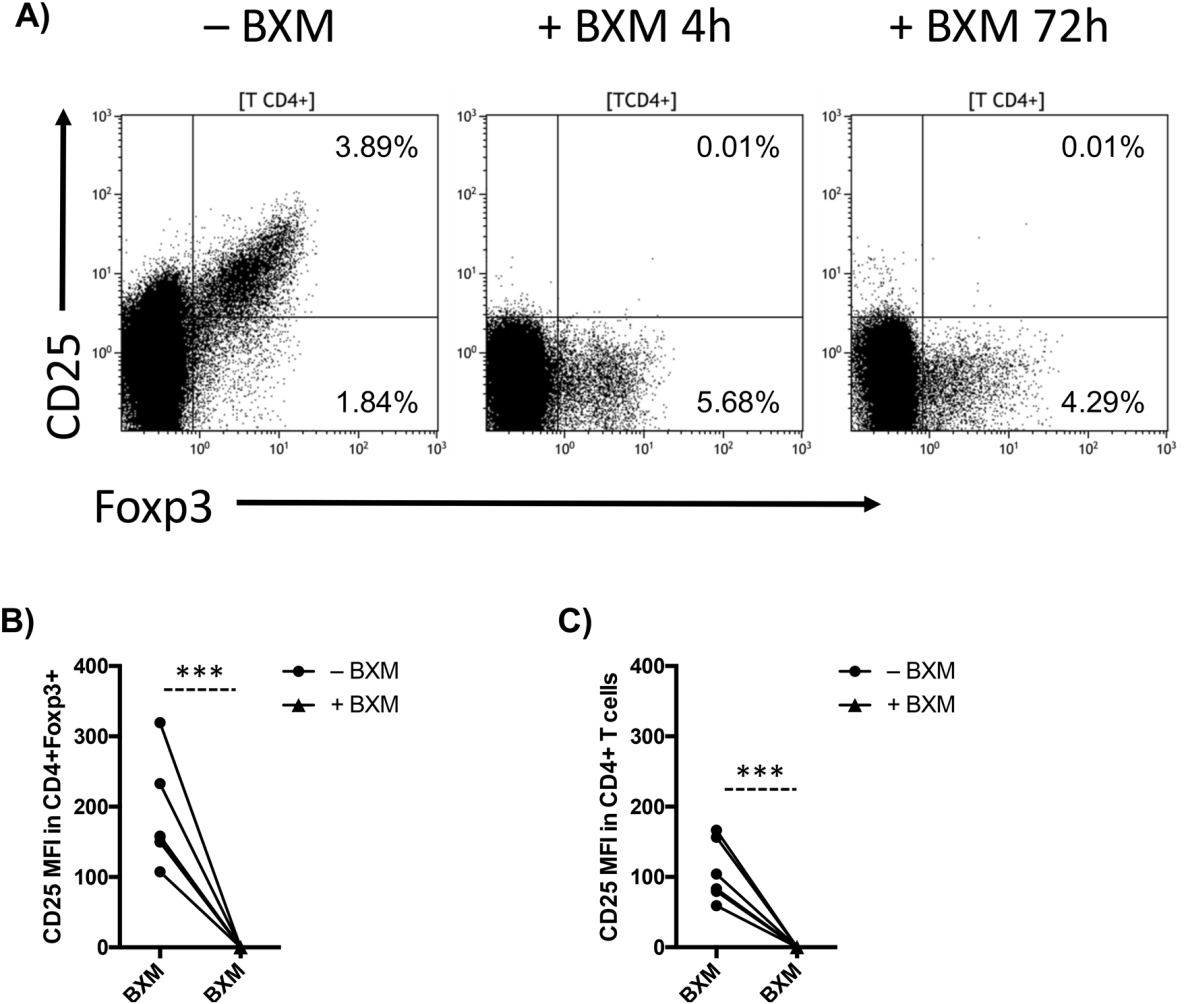
In vitro BXM-dependent CD25 blockade in Tregs. (**A**) CD25 expression within the total CD4+ T-cell population on untreated (-BXM) or BXM treated (+BXM) PBMC from a healthy volunteer after 4 h and 72 h culture. CD25 median fluorescence intensity (MFI) in CD4+ Foxp3+ cells (**B**) and total CD4+ T cells (**C**) within untreated (−BXM, solid circle) or BXM treated (+BXM, solid triangles) stimulated PBMC after 4 h culture (n = 6). ***p < 0.001.

**Figure 2:**
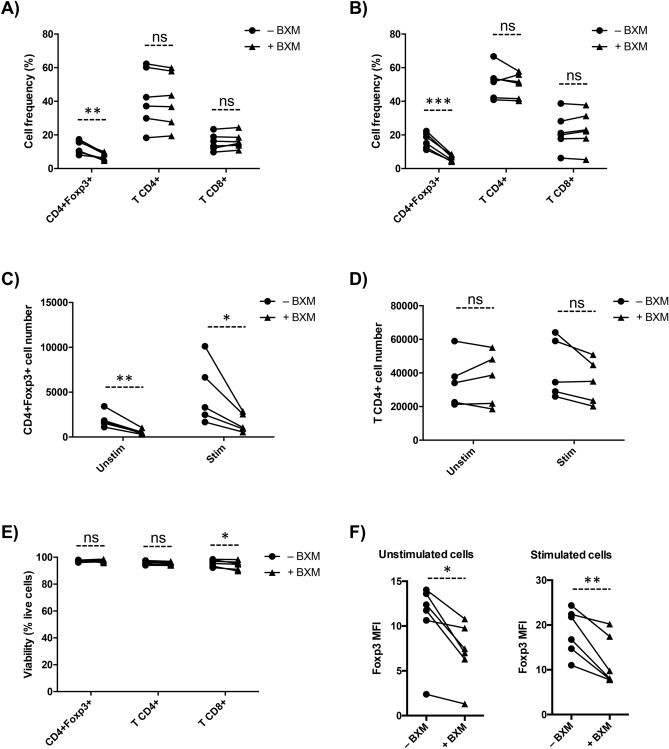
BXM decreases Treg frequency and Foxp3 MFI in vitro. Frequency of CD4+ Foxp3+ (gated on CD4+ T cells), total CD4+ and CD8+ T cells (both gated in lymphocytes) after 72 h culture within PBMC without activation (**A**) or activation (**B**). CD4+ Foxp3+ (**C**) and CD4+ T (**D**) cell number after 72 h in unstimulated PBMC (unstim) or stimulated (Stim) for 72 h. (**E**) Viability after 72 h culture in stimulated PBMC. (**F**) Foxp3 MFI; Each line represents values for untreated (–BXM, solid circles) and BXM-treated (+BXM, solid triangles) conditions for each donor (n = 6). *ns* non-significant; *p < 0.05; **p < 0.01; ***p < 0.001.

To confirm BXM-dependent CD25 blockade in Tregs, we stained cells with a competing anti-CD25 antibody that would be unable to bind CD25 if the receptor is already saturated with BXM^13^. Treatment of PBMC with a single dose of 10 μg/ml BXM completely blocked CD25 in all CD4+ T cells including FoxP3+ Tregs within 4 h of culture, and the blockade remained after 72 h in all six experiments (Fig. 1A–C). With CD25 blocked in BXM-treated cells, we identified the Treg subset via CD4+ Foxp3+ expression. Without activation, BXM induced a marked decrease in Treg frequency (p = 0.003), but no significant effects were observed on total CD4+ (p = 0.282) and CD8+ T cells (p = 0.160) percentages after 72 h culture (Fig. 2A). Similar results were observed when PBMCs were activated (Treg: p < 0.001; T-CD4+ : p = 0.375; T-CD8+ : p = 0.224) (Fig. 2B). We also observed a significant reduction in absolute Treg counts in presence of BXM (approximately 70%; Supplemental Fig. S1A), but not in total CD4+ T-cell counts, in both unstimulated and stimulated conditions after 72 h (Fig. 2C,D). BXM did not induce changes in viability of Tregs (p = 0.687) and total CD4+ T cells (p = 0.123), but there was a trend to decreased viability of CD8+ T cells (p = 0.043) with BXM in stimulating conditions (Fig. 2E). In non-stimulating conditions, BXM treatment did not modify viability of Tregs, or total CD4+ and CD8+ T cells (Supplemental Fig. S1B).

Besides Treg frequency and counts, the Foxp3 expression on Tregs, measured as Foxp3 median fluorescence intensity (MFI) in Foxp3+ CD4+ cells, was significantly reduced after BXM treatment (Fig. 2F) in all six experiments. Reduction of Foxp3 expression was observed in both non-stimulated (p = 0.012) and stimulated conditions (p = 0.006), indicating that BXM also has an impact on Foxp3 expression, the key regulator of Treg function.

### BXM specifically suppresses Treg proliferation in vitro

**Figure 3:**
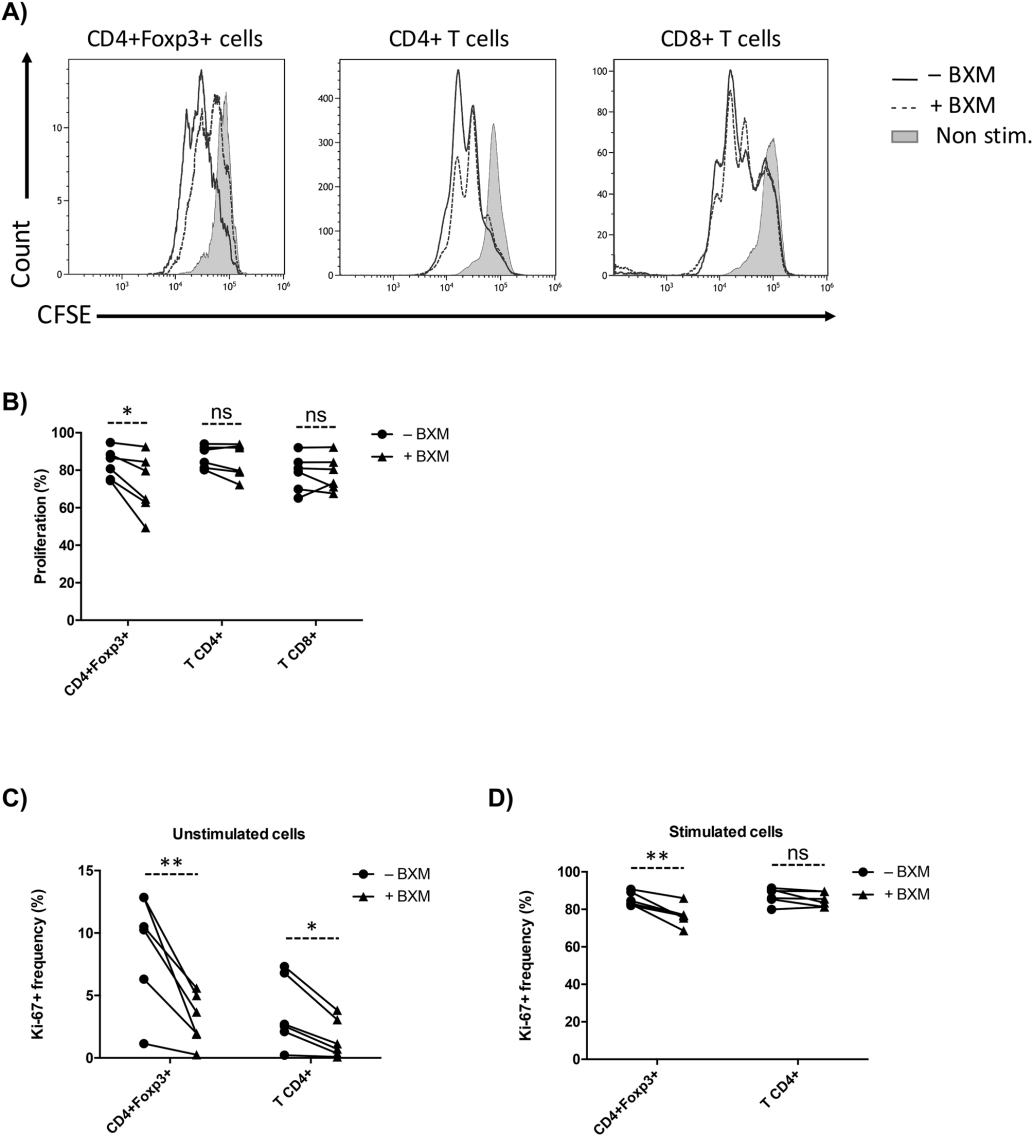
BXM specifically suppresses Treg proliferation in vitro. (**A**) Histograms from a representative donor showing proliferation as a reduction in the CFSE intensity in Tregs, and total CD4+ and CD8+ T cells. (**B**) Values of proliferation based on the loss of CFSE signal (% CFSE lost signal in comparison to CFSE signal on non stimulated cells; Non Stim) in CD4+ Foxp3+, CD4+ and CD8+ T cells within stimulated PBMC. Frequency of Ki-67+ cells on CD4+ Foxp3+ and CD4+ T cells in unstimulated (**C**) or stimulated (**D**) PBMC (n = 6). *ns* non-significant; *p < 0.05; **p < 0.01.

We then investigated the effect of BXM on cell proliferation as a potential mechanism of the observed decrease in Treg proportions. CFSE-labelled PBMC were stimulated and cultured in the presence or absence of BXM. After 72 h we analysed CFSE signal intensity, which decreases upon cellular division. We found that Treg proliferation significantly decreased in the presence of BXM (p = 0.027), while proportions of proliferating cells in total CD4+ (p = 0.204) or CD8+ T cells (p = 0.843) remained unchanged (Fig. 3A,B), indicating that BXM specifically decreases proliferation of the Treg subset.

To further assess the effect of BXM on Treg proliferation, we analysed Ki-67, which is present in all actively dividing cells. In the absence of stimulation (Fig. 3C), BXM induced a pronounced decrease in Ki-67+ frequency in Treg cells (p = 0.008) and a milder decrease in total CD4+ T cells (p = 0.012). In activating conditions (Fig. 3D), BXM induced a significant decrease in Ki-67+ Tregs (p = 0.004) but not in total CD4+ T cells (p = 0.163).

### BXM modifies the cytokine secretion pattern of Treg cells

**Figure 4:**
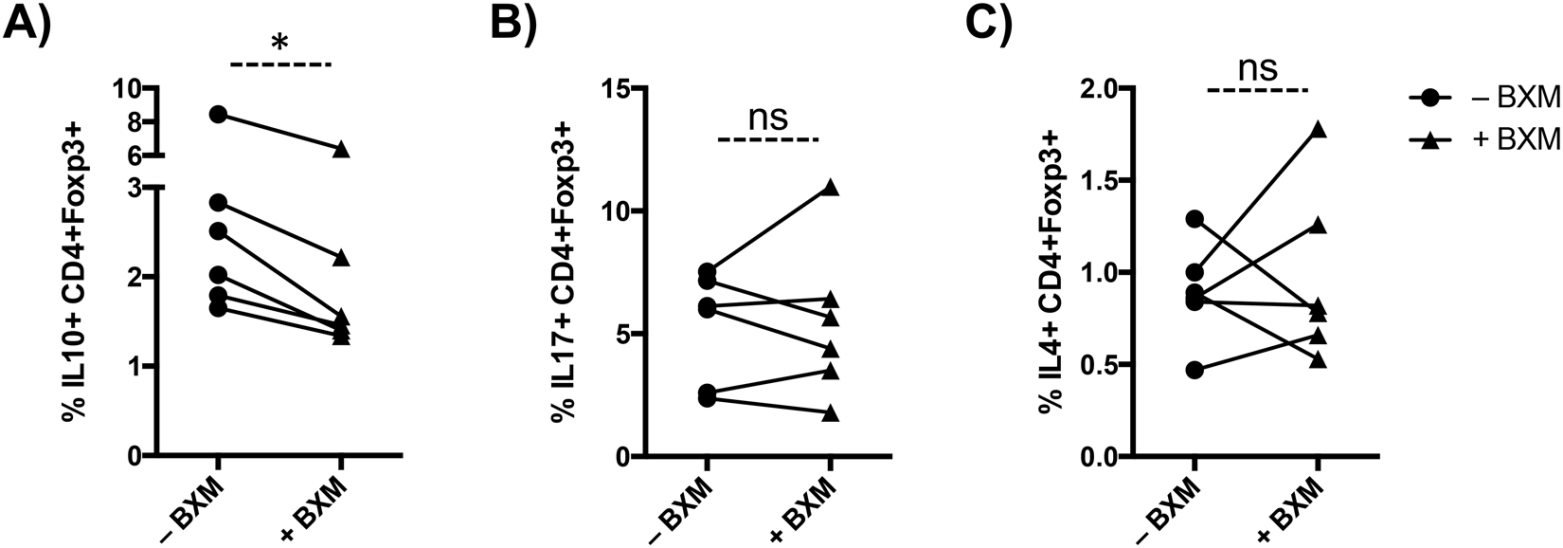
BXM modifies the cytokine secretion pattern of Treg cells in vitro. Percentage of IL-10 (**A**), IL-17 (**B**) and IL-4 (**C**)-secreting cells gated on CD4+ Foxp3+ cells within stimulated PBMC. Each line represents values for untreated (–BXM, solid circles) and BXM-treated (+BXM, solid triangles) conditions for each donor (n = 6). *ns* non-significant; *p < 0.05.

It is known that IL-2 primes Tregs for IL-10 production^14^, and Tregs can be differentiated to Th2-^15^ or Th17-phenotype^16^ after the loss of Foxp3 expression. Because BXM blocked the IL-2 receptor and decreased the Foxp3 expression, we evaluated the cytokine secretion pattern in BXM-treated Tregs. In activated cells, BXM treatment significantly reduced the proportion of IL-10-secreting Treg cells in all six experiments (p = 0.027; Fig. 4A) but did not modify the proportion of IL-4 and IL-17 secreting Tregs (Fig. 4B,C). In non-stimulating conditions, we found an increase in the proportion of IL-10, IL-4 and IL-17 secreting Tregs in BXM-treated cells (Supplemental Fig. S2A–C), suggesting that in a “resting” scenario BXM could also favour a switch of Treg cells into a more pro-inflammatory phenotype and function.

We also examined the expression of inhibitory molecules CTLA-4 and CD39, implicated in Treg suppressive function^17,18^. In both unstimulated and stimulated conditions, BXM did not modify the frequency of CTLA4- or CD39-expressing Tregs (Supplemental Fig. S3).

### Basiliximab temporarily impairs Treg cell population of heart transplant recipients

We investigated whether the detrimental effect of BXM on Treg observed in vitro could be confirmed in vivo by analysing Treg in patients treated or non-treated with BXM.

Six pediatric patients awaiting cardiac transplantation were enrolled in the study (Table 1). The median age of patients was 5.19 years (range 0.14–13.96). In addition to standard immune suppression outlined in the methods, two patients received induction therapy with two doses of BXM (12 mg/m^2^ at day 0 and 4 post-Tx). Patients were clinically followed for at least one year after transplantation. Only *Patient 2* showed signs of cardiac graft rejection (21 days after transplantation); rejection grade 1R with positive C4d staining in the cardiac biopsy confirmed a diagnosis of humoral rejection.

**Table 1:** Characteristics of the patients included in the study.

ID	Sex	Diagnosis	Age at Tx	BXM	Immunosuppression	Pre-sensitized	Rejection episodes	Observations
Patient 1	F	DCM	8 years	Yes	TAC, MMF, Pred	No	No	
Patient 2	F	RCM	14 years	Yes	TAC, MMF, Pred, RIT	No	Humoral—Day + 21	RIT treatment (Day + 26)
Patient 3	M	CC	14 months	No	TAC, MMF, Pred	No	No	
Patient 4	M	DCM	3 years	No	TAC, MMF, Pred	No	No	
Patient 5	M	CC	2 months	No	TAC, MMF, Pred	No	No	
Patient 6	M	RCM	9 years	No	TAC, MMF, Pred	No	No	

*F* female, *M* male, *CC* congenital cardiopathy, *DCM* dilated cardiomyopathy, *RCM* restrictive cardiomyopathy, *Tx* transplantation, *BXM* Basiliximab, *TAC* tacrolimus, *MMF* mofetil mycophenolate, *Pred* metil prednisolone, *RIT* rituximab.

**Figure 5:**
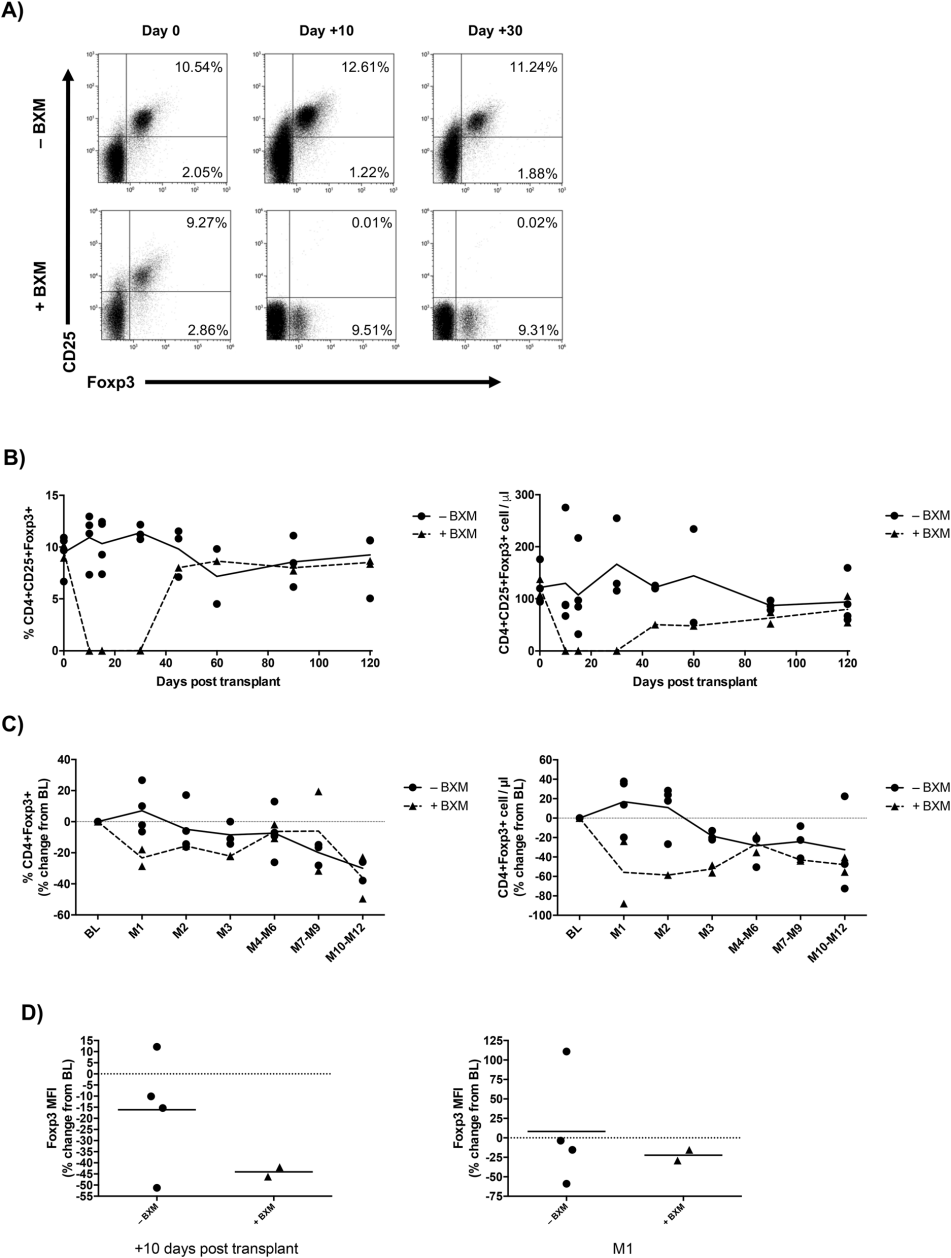
Proportion of CD25+ and or Foxp3+ cells in CD4+ T cells after transplantation. (**A**) Representative dot plots of CD25+ FoxP3+ and CD25-Foxp3+ Tregs (in gated CD4+ T cells). (**B**) Percentages and absolute counts of Tregs from children treated (n = 2; broken line) and non-treated with basiliximab (n = 4; solid line) along 120-days follow-up. (**C**) Percentage of change from baseline values (before Tx; dotted line) for the frequency (left) and absolute counts (right) of CD4+ Foxp3+ T cells from children treated (n = 2; broken line) and non-treated with basiliximab (n = 4; solid line) along 1-year follow-up. (**D**) Foxp3 MFI at day 10 post-Tx (left) and 1-month post-Tx (right). Dotted line represents the baseline values before Tx and solid lines the mean values along the follow-up.

Tregs at Day-0 were identified as CD4+ Foxp3+ CD25+ (Fig. 5A); all enrolled patients showed normal Treg values before transplantation when compared to those previously published by Schatorjé et al.^19^. Ten days after introduction of immune suppressive therapy, patients not treated with BXM showed preserved proportions of CD25^+^Foxp3^+^ Tregs. Nevertheless, as observed in our in vitro experiments (Fig. 1), the epitope recognized by the anti-CD25 antibody used to detect these Treg cells was completely blocked in BXM-treated patients. This BXM-mediated CD25 blockade in CD4+ Foxp3+ cells was evident for at least 30 days; CD25 was detectable by 45 days post-transplant (post-Tx) (Fig. 5A,B left panel), which corresponds to BXM peripheral clearance^20^. From Day + 45 until 4 months post-Tx, Foxp3+ CD25+ Treg frequencies in the BXM group remained stable and comparable to those of BXM-untreated patients. However, Treg absolute counts (cells per μl of blood) did not reach pre-transplant levels after BXM treatment and remained low compared to the BXM-untreated group up to 3 months post-Tx (Fig. 5B right panel).

We followed the evolution of the Treg subset over time independently of their CD25 availability, analysing all (CD25+ and CD25−) CD4+ Foxp3+ cells (Fig. 5C). Compared to baseline values, we observed a slight decrease in Treg proportion in BXM-treated patients during the first 3 months after Tx, which was more pronounced in terms of Treg absolute counts (cells per µl of blood, right panel), where we could observe a decrease in Treg counts up to 50%. Of note, the recovery of CD25 availability after BXM clearance (day 45) was not immediately followed by significant recovery in the numbers of CD4+ Foxp3+ cells in BXM-treated patients, which seem to reach normal levels after 3 months post-Tx.

Moreover, as observed in our in vitro setting (Fig. 2F), BXM treatment also induced a reduction of Foxp3 expression in patients’ cells (Fig. 5D). At Day + 10 post-Tx, in 3 of the 4 patients who did not receive BXM, their Foxp3 MFI remained close to baseline levels. However, the two BXM-treated patients showed a huge decrease (> 40%) in Foxp3 MFI at Day + 10, which was still present, but less pronounced, 1-month post-Tx.

Additionally, we assessed the impact of BXM induction therapy on immune parameters related to potential risk of graft rejection. BXM-treated patients showed a trend to increased values of both activated and effector memory CD8+ T-cell over the 1-year follow-up, whereas no differences were found in CD4+ T-cells (Supplemental Fig. S4A–D). Interestingly, patients treated with BXM showed lower mean ratios of Treg/CD4Teff and Treg/CD8Teff than patients not treated with BXM over the 12-month follow-up (Supplemental Fig. S4E,F).

Altogether, these observations indicate that BXM treatment could be negatively affecting to Treg cells. Even the transitory of this affection, it could in part be responsible for the increased values of activated and memory CD8+ T-cells that can promote the graft rejection in medium-long term. Although the low number of patients analysed per group hindered the drawing of any robust conclusion, they are in line with our in vitro findings about the effects that BXM could have on the Treg cell population, which has been proved to have a key role in graft protection.

## Discussion

Tregs have been shown to play a crucial role in preventing early graft rejection, and there is evidence suggesting that Tregs could facilitate long term graft tolerance in transplantation^21,22^. However, the mechanisms involved in graft acceptance could be compromised by the use of certain immunosuppressive drugs. Since CD25+ Tregs share the target of BXM with the cell populations mediating rejection, it became crucial to assess the balance of the drug effect between targeting effector cells and not hampering Treg functionality and survival.

In our in vitro study, we treated human PBMC with BXM and observed a decrease in CD4+ FoxP3+ frequency and absolute numbers, indicating a depletion of Treg cells. Since we did not observe direct effect of BXM on Treg viability, we dismissed the possibility that the decrease in Tregs could be due to increased Treg mortality from higher toxicity of BXM in these cells. This is consistent with the findings of Wang and colleagues, who demonstrated that in the presence of BXM, CD4+ CD25+ T cells were not depleted from the circulating pool through monoclonal antibody activation-associated apoptosis^23^. In our attempt to identify specific mechanism(s) responsible for the BXM-mediated Treg depletion, we demonstrated that BXM treatment markedly decreases Treg proliferation. CFSE-labelling of human PBMCs can account for up to 50% cell death and modifies activation markers^24^, and for that we further confirm the BXM effect on Treg proliferation analysing Ki-67, which is present in all actively dividing cells. Moreover, BXM-mediated impairment of Treg proliferation was observed in both unstimulated and stimulated cells. This indicates a potential effect of BXM on the homeostatic proliferation of Treg in a quiescent state, but also on Treg proliferation in response to expansion of effector cells that could mediate graft rejection. Our in vitro findings are in agreement with the results reported by Bouvy et al. in kidney transplant recipients, reporting that patients receiving rabbit antithymocyte globulin (rATG) induction therapy had higher percentages of Ki-67+ Tregs after 1 month than before transplantation whereas patients with BXM induction had very low percentages of Ki-67+ Tregs^25^.

In contrast to the high impact on Tregs, total CD4+ T-cell frequency and proliferation capacity were not affected in vitro by BXM, suggesting that BXM may have stronger effects on Tregs compared to the total CD4+ population, the latter being the main desired target of this drug. This is likely related to higher demand for IL-2 by Tregs to maintain proliferation, as shown in previous studies where in contrast to Treg, non-Treg cells have been shown to proliferate upon antigen stimulation in an IL-2 deficient environment^26^.

We also have the unique opportunity of analyzing the in vivo effect of BXM in a small cohort of pediatric heart transplant patients (two receiving BXM induction therapy and four without BXM induction). In our study, CD25-blockade in Tregs from pediatric patients lasted for 30–45 days, which matches the timeframe of basiliximab elimination in serum reported in pediatric liver and kidney transplant patients^20,27^. Independently of CD25 availability, BXM-mediated IL-2 signalling deprivation appears to cause a decrease in Treg (Foxp3+ CD4+ cells) numbers in comparison to BXM-untreated patients, which persists for at least four months. This is contradictory with results provided by several authors^23,27–29^, showing that frequency of Foxp3-expressing CD4+ T cells remains unchanged in transplanted patients treated with BXM. All these articles analysed percentages of Foxp3+ cells, which is a relative frequency that could be influenced by changes in other subsets of T cells. To avoid this, we analysed the absolute counts of Foxp3+ cells per μl of blood, a better indicator of the true number of cells available in the periphery.

The effect of anti-CD25 on Treg in heart transplant adults^30^ and the relevance of the Treg subset in rejection in these heart transplant recipients has been previously described^31^. However, in contrast to other studies, our cohort of heart transplant pediatric patients was thymectomized, and the regeneration and production of new Tregs are likely seriously compromised in these patients. Because in these patients the capacity of T-cell replenishment is compromised by the absence of a functional thymus, the effect of BXM on Treg counts could be irreversible or having more serious consequences than in other pediatric patients, in which the thymus is intact, or than in adult patients where the whole T-cell repertoire has been correctly generated in the infancy. The fact that Treg frequency and absolute numbers were more affected by BXM than total CD4+ and CD8+ cells had a strong impact on Treg/Teff ratio, which was shown to be critical for rejection freedom^32,33^. BXM-treated patients showed lower Treg/Teff CD4+ and CD8+ T cells, notably in the first months after BXM treatment, a pattern described to be less favourable for graft acceptance.

Due to the very low number of heart transplants performed in children per year it was not possible to enrol a large enough sample to draw definitive conclusions, but these clinical observations reflect somehow our in vitro results, which provided objective evidence of the detrimental effect of BXM on Treg values and phenotype. Presence of BXM in concentrations comparable to the serum concentration that is reached in pediatric patients treated with BXM^20^ produced a complete blockade of CD25, which it was related with a decrease in the frequency and number of Tregs.

We also observed in vitro and in vivo that BXM-mediated IL-2 signalling deprivation induces a clear decrease in Foxp3 expression on Tregs, which may impair their suppressive capacity^34^. This finding agrees with other studies in the context of anti-CD25 antibody treatment of multiple sclerosis^35^ and our previous studies showing altered levels of CD25 affecting Foxp3 expression^36^. Foxp3 downregulation in Tregs has also been associated with a switch to a secretion pattern of pro-inflammatory cytokines^15,16,34^. In stimulated conditions, which could mimic the Treg response in a scenario of immune activation against the graft, IL-4 and IL-17 secretion by Treg was not modified with BXM, but a clear reduction was observed in the frequency of IL-10-producing Tregs. This is consistent with the necessity of IL-2 signalling to prime IL-10 production in Tregs^14^, and may also be related to Foxp3 downregulation^15,37^.

If one translates these results to a physiological context, we could hypothesize that if an immune response against alloantigens is initiated in the presence of BXM, Treg proliferation will not match the proliferation of CD4+ and CD8+ cells, which may lead to a decrease in the ratio between Treg/Teff with potential negative impact on graft acceptance. This hypothesis is supported by our findings that BXM has a stronger suppressive effect on Treg proliferation in activation conditions, which will render the immune system having a lower Treg number to neutralize T-cell effector proliferation. In addition to their decreased numbers and the imbalance with effector cells, Tregs will undergo Foxp3 downregulation and a reduction in their capacity to secrete IL-10, which contrasts with other articles reporting that BXM does not impair Treg suppressive functions (reviewed in^38^).

We cannot exclude that other immunosuppressants administered as maintenance therapy may also have detrimental effects on Tregs. In fact, calcineurin inhibitors such as TAC inhibit IL-2 production, and this could also interfere with the number and function of Tregs^39^. However, considering the controversy about the risks/benefits of BXM induction, the minimal effect of BXM decreasing the absolute risk of acute rejection reported by several authors^40–42^, together with the detrimental effect on Tregs shown in our study, cast doubt on the overall usefulness of BXM in pediatric heart transplantation. A recent large registry study employing multivariable models investigated the benefits of induction therapy in a cohort of pediatric heart transplanted patients after risk stratification^43^. Castleberry et al*.* report that, although use of anti-CD25 induction therapy was associated with lower rates of rejection and infection compared to no induction, and overall graft survival was higher in patients who received induction therapy, a clear relationship between survival and use of induction therapy could not be proven, based on multivariable analysis.

Besides better preservation of Tregs, we have observed (in a reduced cohort of 4 patients) that transplantation without BXM does not result in increased effector or activated CD4+ or CD8+ T cell populations that could potentially promote graft rejection. In fact, *Patient 2* (BXM-treated) did not have donor directed antibodies before transplant but had humoral rejection at Day + 21 post-Tx. *Patient 2* was the patient with lower Treg counts after the first month post-Tx who had increased values in both effector memory and activated CD8+ cells from Day + 90 onwards.

In conclusion, our findings demonstrate a detrimental effect in vitro of BXM on Tregs, impairing their number and function by affecting their proliferative capacity, Foxp3 expression, and IL-10 secretion capacity. Due to the small number of pediatric heart transplant patients available, we cannot extrapolate these conclusions to the in vivo context, but they reinforce the results obtained in vitro*,* suggesting that detrimental effects of BXM on Tregs are probably produced in vivo, which could negatively affect the protective role of Treg populations just in the period of highest incidence of graft rejection^44^. Therefore, BXM effects on Tregs and immune homeostasis must be considered in the design of appropriate immunosuppressive regimens for these patients.

## Patients and methods

### Human samples

Fresh blood samples were obtained from pediatric heart transplant patients (n = 6) either treated (n = 2) with basiliximab (BXM, Simulect, Novartis Pharma, Basel, Switzerland) in a dose of 12 mg/m^2^ on Days 0 and 4 after transplantation (Tx) or without BXM (n = 4). All patients received maintenance immunosuppressive therapy consisting of short-term steroids, mycophenolate mofetil (MMF) and tacrolimus (TAC). None of the patients were pre-sensitized, they did not have donor directed HLA antibodies before transplant. *Patient 2,* who showed signs of rejection at day + 21 post-Tx, received three boluses of methylprednisolone together with intravenous immunoglobulin (IVIG), and four weekly doses of rituximab (375 mg/m^2^ per week) from day + 26 post-Tx (Table 1). Rituximab is a chimeric monoclonal antibody that specifically binds to the CD20 molecule, the expression of which is restricted to B cells. Therefore, we considered that it would not have any direct effect on Treg cells, and even has been described that it could indirectly increase the Treg levels^45^.

The study was conducted after approval of the ethics committee of *Hospital Gregorio Marañón* (Madrid, Spain) and according to the principles expressed in the Declaration of Helsinki. Written informed consents from the legal guardians were obtained before patient´s enrolment. All the samples were obtained at the Pediatric Cardiology Unit of the *Hospital Gregorio Marañón*. Peripheral blood samples (< 3 ml) were drawn before of transplantation (Day-0 or BL) and at 10, 15, 30, 45, 60, 90, 120, 180, 270, 360 days post-Tx. Baseline samples (BL or Day-0), were obtained 1–2 days before the transplant procedure and the administration of immunosuppressive therapy, including BXM. Fresh blood samples were always processed within 2 h after extraction. Peripheral blood mononuclear cells (PBMC) employed for in vitro experiments were obtained from buffy coats of adult subjects from the Transfusion Centre of Madrid.

### Culture and in vitro treatment of PBMCs

PBMCs from buffy coats were isolated on a Ficoll-Hypaque (Rafer, Madrid, Spain) density gradient and treated with BXM (Simulect, Novartis Pharma, Basel, Switzerland) to analyze its effect on CD4, CD8 and Treg viability, proliferation, Foxp3 expression and cytokine secretion. Briefly, CFSE-labeled PBMCs (CFSE from Life Technologies, Carlsbad, CA) were cultured with RPMI 1640 medium (Biochrome) supplemented with 10% heat-inactivated FCS, 500 U/ml IL-2 and a mix of antibiotics (125 µg/ml cloxacillin, 125 µg/ml ampicillin and 40 µg/ml gentamicin; Sigma-Aldrich, St. Louis, MO). CFSE-labeled PBMCs (Life Technologies) were stimulated with anti-CD3/anti-CD28-coated beads (Life Technologies) at 0.5:1 or 1:1 ratio (bead:PBMC), treated with 10 µg/ml of BXM (Simulect, Novartis Pharma) and cultured for 72 h in the presence of IL-2. This concentration was chosen considering the serum concentration that is reached in pediatric patients treated with BXM^20^, and looking for maximal suppressive effect^46^. After culture, all samples were analysed by flow cytometry.

### Analysis of immune subsets by flow cytometry

The frequency and absolute counts of different T-cell subsets were analysed in peripheral blood from patients using a combination of specific antibodies as previously described^47,48^. Briefly, we determined by flow cytometry (*Gallios Cytometer*, Beckman Coulter, France) the frequency and absolute counts of CD4+ T cells and CD8+ T cells, including markers for the following subsets: activated (HLA-DR+) and effector memory (CD45RA− CD27−). Absolute counts and frequency of Treg cells in peripheral blood were quantified by measuring either CD3+ CD4+ CD25+ Foxp3+ or CD3+ CD4+ Foxp3+ cells. Of note, anti-CD25 clones BC96 (Ebioscience. San Diego, CA) and 2A3 (Becton Dickinson, Franklin Lakes, NJ) were used, these clones compete unsuccessfully with BXM for CD25 binding^13^, enabling us to track BXM-dependent CD25 blockade by the absence of anti-CD25 signal. Foxp3, together with CTLA-4 and Ki-67 were analysed by intracellular staining with the *Anti-Human Foxp3 Staining Set* (Ebioscience) according to the manufacturer’s instructions. Activated and effector memory subsets of Treg cells were also analysed. Absolute numbers of immune subtypes were determined using *Flow-Count Fluorospheres* (Beckman–Coulter).

### Analysis of cytokines

Analysis of cytokine-secreting Treg cells was performed in viable PBMCs (identified by using Fixable Viability Dye eFluor 450, Ebioscience). Briefly, PBMCs were activated for 5.5 h with PMA (50 ng/ml) and Ionomycin (1 ug/ml), including the addition of Brefeldin A (reagents from Sigma-Aldrich, St. Louis, MO). Intracellular staining of Foxp3, IL-10, IL-4 and IL-17 was performed following instructions of the Anti-Human Foxp3 Staining Set (Ebioscience). The frequency of cytokine-secreting cells was calculated in the CD4+ Foxp3+ population.

### Statistical analysis

Statistical analysis was performed using SPSS Software (IBM). T-student test for paired samples was used for comparison between treated and untreated conditions in each of the in vitro experiments. Because of the small patient number in the BXM-treated group (n = 2) no statistical analysis was performed for the in vivo study.

## Supplementary Information

Supplementary Information.

## Abbreviations

BXM: Basiliximab
CFSE: Carboxyfluorescein succinimidyl ester
CTLA-4: Cytotoxic T-lymphocyte antigen 4
Foxp3: Forkhead box protein 3
IL-2Ra: IL-2 receptor alpha chain
IL-10: Interleukin 10
IL-17: Interleukin 17
IL-2: Interleukin 2
IL-4: Interleukin 4
IVIG: Intravenous immunoglobulin
MFI: Median fluorescence intensity
MMF: Mycophenolate mofetil
PBMC: Peripheral blood mononuclear cells
PMA: Phorbol myristate acetate
Post-Tx: Post transplant
TAC: Tacrolimus
SEM: Standard error of the mean
Teff: Effector T cells
Th1: T-helper 1
Th2: T-helper 2
Tregs: Regulatory T cells
