# Supplementary material for: Basiliximab impairs regulatory T cell (TREG) function and could affect the short-term graft acceptance in children with heart transplantation
Source: Sci Rep. 2021 Jan 12;11:827. doi: 10.1038/s41598-020-80567-9 (PMC7803770; doi:10.1038/s41598-020-80567-9)
Supplement: Supplementary file 1 — Supplementary Information. [file 41598_2020_80567_MOESM1_ESM.pdf]

SUPPLEMENTARY TABLES AND FIGURES

BASILIXIMAB IMPAIRS REGULATORY T CELL (TREG) FUNCTION AND COULD AFFECT THE SHORT-TERM GRAFT ACCEPTANCE IN CHILDREN WITH HEART TRANSPLANTATION

Jacobo López-Abente, PhD[a]; Marta Martínez-Bonet, PhD[a]; Esther Bernaldo-de-Quirós[a]; Manuela Camino, MD[b]; Nuria Gil, MD[b]; Esther Panadero, MD[b]; Juan Miguel Gil-Jaurena, MD-PhD[c]; Maribel Clemente, PhD[d]; Simon Urschel, MD-PhD[e,f,g]; Lori West, Md-PhD[e,f,g]; Marjorie Pion, PhD[a]; Rafael Correa-Rocha, PhD[a,g] *

[a]Laboratory of Immune-regulation, *Instituto de Investigación Sanitaria Gregorio Marañón* (IiSGM), Madrid (Spain); [b]Pediatric-Cardiology and [c]Pediatric Cardiac Surgery Units, *Hospital General Universitario Gregorio Marañón, Madrid (Spain);* [d]Cell Culture Unit, *Instituto de Investigación Sanitaria Gregorio Marañón* (IiSGM), Madrid (Spain); [e]Pediatric Cardiac Transplantation, University of Alberta/Stollery Children's Hospital, Edmonton, AB, Canada; [f]Alberta Transplant Institute, University of Alberta, Edmonton, AB, Canada; [g]Canadian National Transplant Research Program investigator, CNTRP, Edmonton, AB, Canada.

***Corresponding Author*: Rafael Correa-Rocha, PhD**
Email: rafael.correa@iisgm.com; ORCID: 0000-0003-3456-9986

Laboratory of Immune-regulation. Instituto de Investigación Sanitaria Gregorio Marañón.
Pabellón de Medicina Experimental. Planta Baja. C/ Maiquez, 6. 28006 Madrid (SPAIN)
Phone: +34 915866455

Supplemental Figure 1

A)

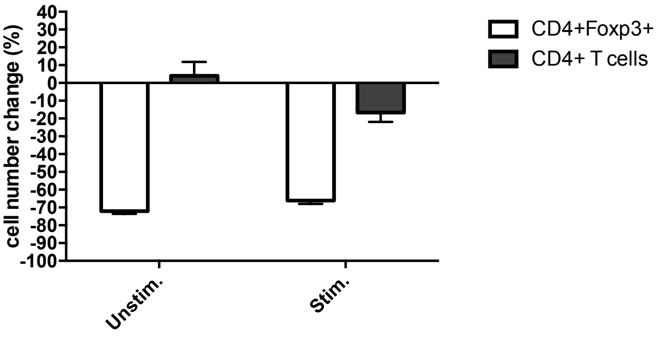

B)

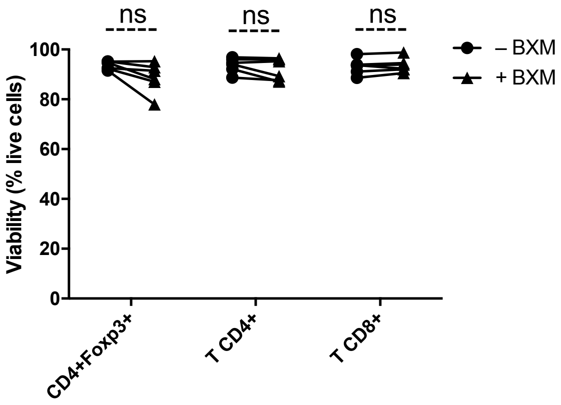

SUPPLEMENTAL FIGURE 1. (A) Graph showing the variation (expressed as %) in the absolute counts of CD4+Foxp3+ (white bars) and CD4+ T cells (grey bars) after 72h hours culture with BXM within PBMC without stimulation (Unstim) or stimulated (Stim) with anti-CD3/CD28 dynabeads at 0.5:1 (bead:cell ratio). (B) Viability after 72h culture in CD4+Foxp3+, CD4+ and CD8+ T cells within un-stimulated PBMC. Each line represents values for untreated (–BXM, solid circles) and BXM treated (+BXM, solid triangles) conditions for each donor, n = 6. ns: non-significant statistical differences between untreated (–BXM) and treated (+BXM) condition in T-test for paired samples. ns= non-significant

Supplemental Figure 2

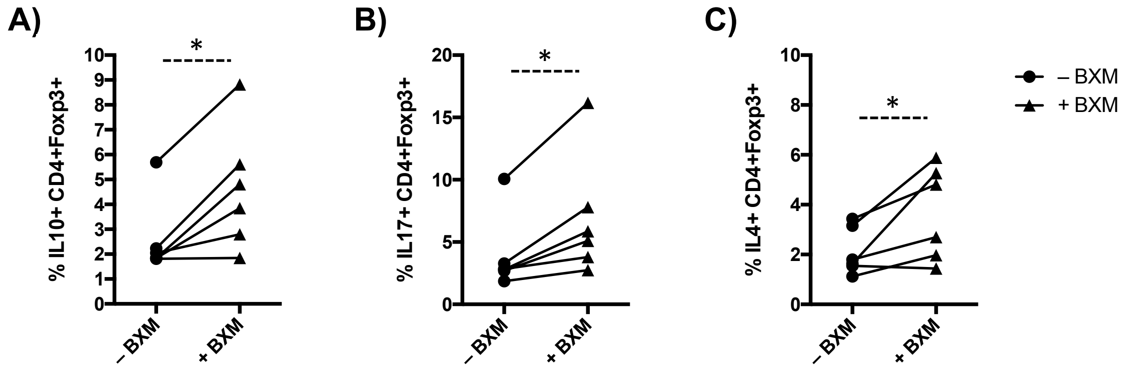

SUPPLEMENTAL FIGURE 2. BXM effect on cytokine secretion by Treg. Graph showing the % of IL-10 (A), IL-17 (B) and IL-4(C)-secreting cells gated on CD4+Foxp3+ cells within non-stimulated PBMC after 72h culture. Each line represents values for untreated (–BXM, solid circles) and BXM treated (+BXM, solid triangles) conditions for each donor, n = 6. *: $p<0.05$ statistical differences between untreated and treated condition in T-test for paired samples. * $p<0.05$

Supplemental Figure 3

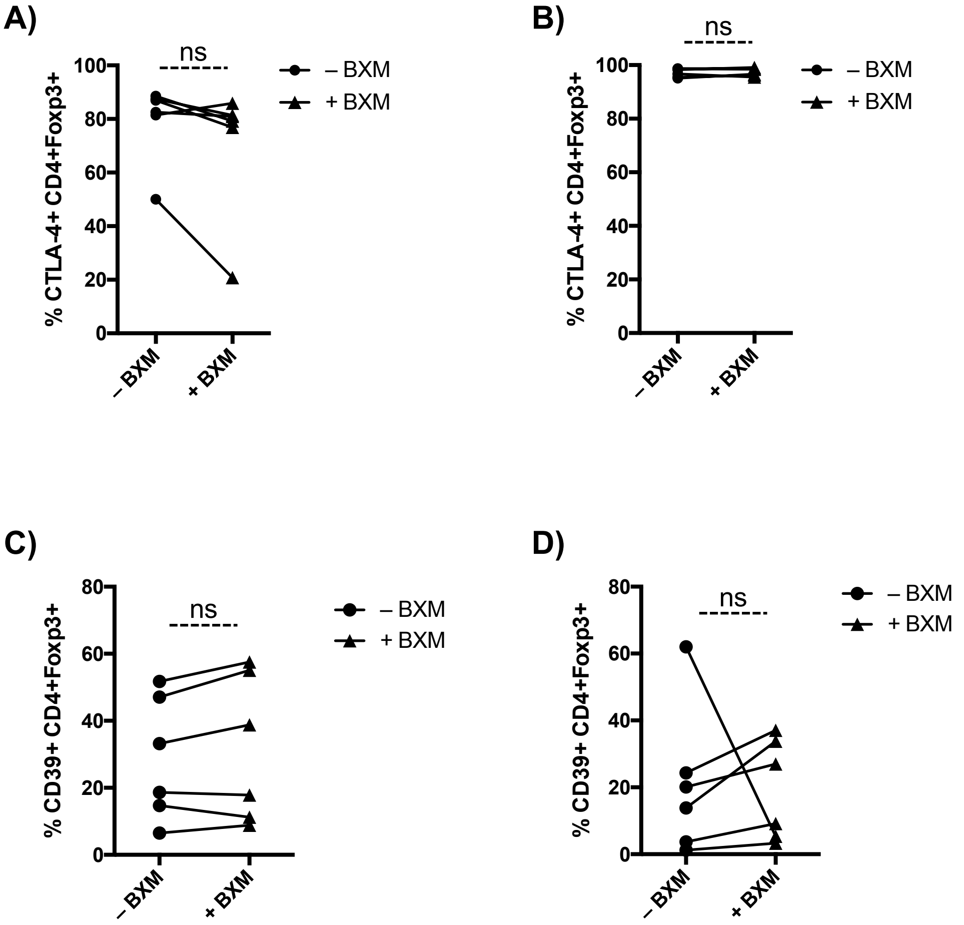

SUPPLEMENTAL FIGURE 3. BXM effect on Treg phenotype. Graph showing the percentage of CTLA-4+ gated on CD4+Foxp3+ cells within non-stimulated (A) and stimulated PBMC (B). Frequency of CD39+ gated on CD4+Foxp3+ cells within non-stimulated (C) and stimulated PBMC (D). Each line represents values for untreated (–BXM, solid circles) and BXM treated (+BXM, solid triangles) conditions for each donor, n = 6. ns: non-significant statistical differences between untreated and treated condition in T-test for paired samples. ns= non-significant

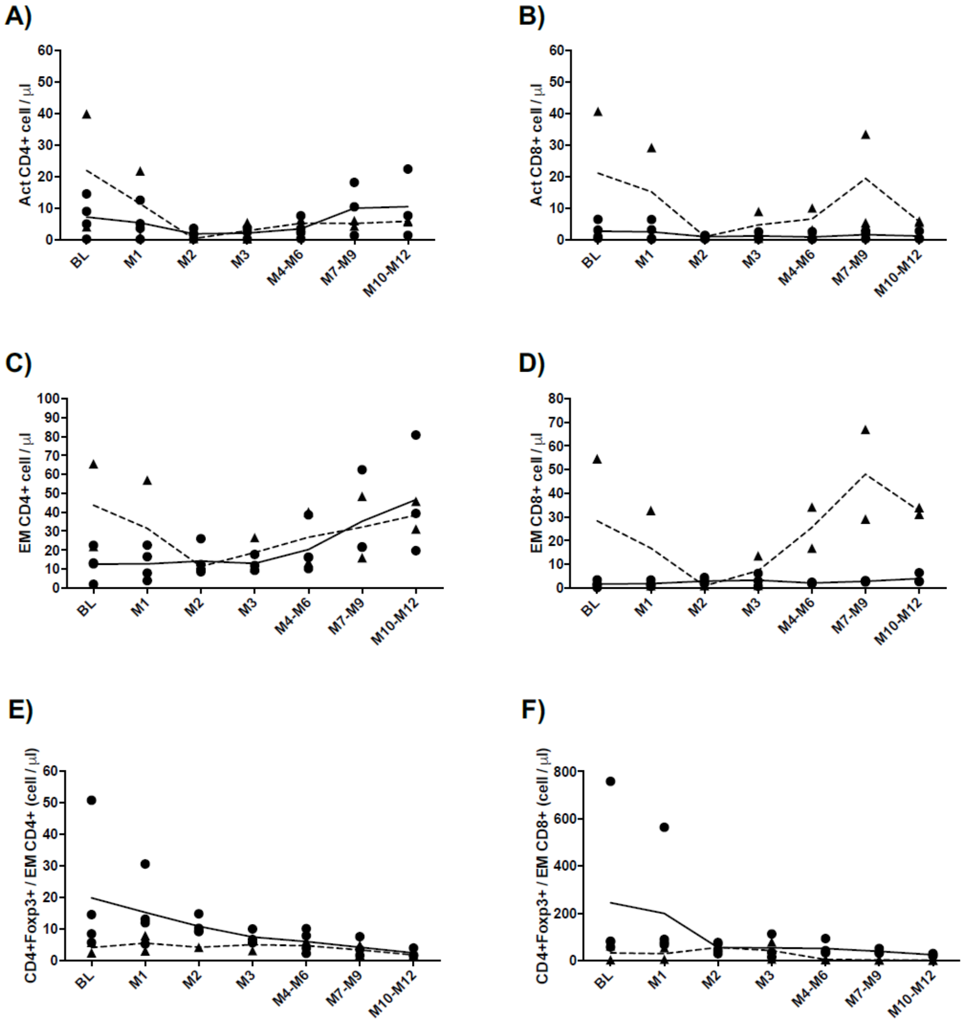

SUPPLEMENTAL FIGURE 4. Impact of BXM induction therapy on immune parameters related to potential risk of graft rejection. Absolute counts of activated (Act) CD4+ (A) and CD8+ (B) T cells. Absolute counts of effector (EM) CD4+ (C) and CD8+ (D) T cells. Ratio between counts of Foxp3-expressing Tregs and effector CD4+ T (E) or CD8+ T cells (F). Individual values for children treated (n=2; broken line) and non-treated with basiliximab (n=4; solid line) along 1-year follow-up, and a line representing mean values in each group are represented.
